# Short-Term Effects of Verapamil and Diltiazem in the Treatment of No Reflow Phenomenon: A Meta-Analysis of Randomized Controlled Trials

**DOI:** 10.1155/2015/382086

**Published:** 2015-10-04

**Authors:** Lan Wang, Zhong Cheng, Ye Gu, Dingfeng Peng

**Affiliations:** Heart Center at Puai Hospital, Wuhan Puai Hospital, Huazhong University of Science and Technology, Wuhan 430030, China

## Abstract

Currently, there is still a lack of an optimal treatment for no reflow phenomenon (NRP). We analyzed the efficacy and safety of using nondihydropyridine calcium channel antagonists (NDHP, verapamil/diltiazem) in patients suffering from NRP. Eight RCTs with 494 participants were eligible for analysis. The pooling analysis showed that intracoronary verapamil/diltiazem injection significantly decreased the occurrence of the coronary NRP (RR: 0.3, 95% CI: 0.16–0.57; *P* = 0.0002) and reduced corrected thrombolysis in myocardial infarction (TIMI) frame Count (WMD = −9.24, 95% CI −13.91–4.57; *P* = 0.0001) in patients with NRP. Moreover, verapamil/diltiazem treatment showed superiority in reducing wall motion index (WMI) compared to the control at day 1 (WMD = 0.11, 95% CI: 0.02–0.20; *P* = 0.02) (*P* < 0.05). There was also a significantly greater decline at occurrence of the major adverse cardiac events between verapamil/diltiazem and control groups (WMD: 0.4, 95% CI: 0.19–0.84; *P* = 0.02). However, using verapamil/diltiazem did not provide additional improvement of left ventricular ejection fraction post procedure (at 7 days, WMD, 0.1; 95% CI, −2.43–2.63; *P* = 0.94; at 30 days, WMD, 0.42; 95% CI, −2.09–2.92; *P* = 0.75). NDHP use is beneficial in attenuating NRP and reducing 6-month MACEs in patients with NRP.

## 1. Introduction

No-reflow phenomenon is defined as inadequate myocardial perfusion of the adequately dilated target vessel without evidence of angiographic mechanical obstruction [1], angiographic no flow refers to TIMI 0-1 and slow flow refers to TIMI 2 [2], and no reflow and slow flow phenomenon have been observed in 0.6% to 2% of patients receiving percutaneous coronary interventions (PCIs) [3–6]. Incidence of angiographic no reflow phenomenon has been reported as high as 10% to 44% in acute myocardial infarction (AMI) patients undergoing PCI [7, 8]. Clinical studies revealed that patients exhibiting no/slow reflow following reperfusion therapy ischemic heart disease were associated with worse prognosis compared to patients without no-flow [4, 6].

Key pathogenic components of no/slow flow phenomenon include distal atherothrombotic embolization, ischemic injury, reperfusion injury, and susceptibility of coronary microcirculation to injury [9]. Thus, pharmacologic and mechanical strategies to prevent and treat no reflow also target these mechanisms; pharmacologic therapy with vasodilators and antiplatelet agents as well as mechanical therapies with distal protection and aspiration thrombectomy have shown benefit in the treatment of no/slow reflow phenomenon [10]. Although local vasodilator therapy and local antiplatelet therapy are widely used to manage the clinical no/slow phenomenon, only local vasodilator therapy has a specific guideline indication for treatment of no reflow and The 2011 ACC PCI guidelines [11] give a class IIa recommendation for administration of an intracoronary vasodilator (specifically, adenosine, calcium channel blocker, or nitroprusside) to treat PCI-related no reflow that occurs during primary or elective PCI.

Efficacy of intracoronary nondihydropyridine calcium channel antagonists (NDHP), verapamil/diltiazem, administration during PCI for the management of reduced coronary flow due to microvascular dysfunction was already evidenced in the 1990s in selected patients [12, 13]. Previous studies also showed that NDHP significantly improved coronary and myocardial perfusion when given prophylactically prior to elective PCI [14, 15] and in patients undergoing primary PCI for AMI [16]. Recently, Su et al. analyzed the short-term effect of verapamil [17] as well as adenosine and verapamil [18] on coronary no reflow associated with percutaneous coronary intervention in patients with acute coronary syndrome. Present meta-analysis focused on the efficacy and safety of verapamil/diltiazem use for the management of no/slow phenomenon.

## 2. Methods

### 2.1. Search Strategy

We search the PubMed, OVID, EBSCO, and Cochrane Library (1980–2015) to identify randomized controlled trials (RCTs) and observational studies. Searches were conducted without any language restrictions using the key words “slow flow,” “no reflow,” “verapamil,” and “diltiazem”.

### 2.2. Study Selection

The selection criteria for inclusion in this meta-analysis were as follows: RCTs studies that randomly allocated patients with coronary no reflow or slow flow to undergo intracoronary/oral infusion of verapamil/diltiazem or placebo/blank control; studies reported at least on one of following outcomes: thrombolysis in myocardial infarction (TIMI) flow as well as TIMI frame count (CTFC), major adverse cardiac events (MACEs, such as all-cause death, target vessel revascularization, recurrent angina or myocardial infarction, and severe heart failure), left ventricular ejection fraction (LVEF), or wall motion index (WMI).

### 2.3. Data Extraction

Data extraction was performed by two independent reviewers using a standardized data-collection form, and disagreements were resolved by discussion. Patient characteristics, study quality, and clinical outcomes including CTFC, TIMI flow, MACEs, LEVF, and WMI were analyzed in both NDHP and control groups.

### 2.4. Methodological Quality Assessment

We evaluated trials for concealment of treatment allocation, clear description of the design, and completeness of follow-up. The JADAD scale [26] was used to score study quality (range of 0–5, higher scores indicating higher quality). Quality assessment was undertaken independently by two reviewers.

### 2.5. Statistical Analysis

Results were analyzed with Review Manager 5.2 software (Cochrane Collaboration, Oxford, England). Egger's asymmetry test was performed using Stata13.0 software (available from http://stata.com/). Heterogeneity was tested using the *χ* ^2^ test (with *P* < 0.05 indicating significant heterogeneity). The meta-analysis was performed using the fixed effects model if *P* > 0.05; otherwise, the random effect model was used. Odds ratios (ORs) were calculated for dichotomous variables and the weighted mean difference (WMD) was calculated for continuous variables. Summary statistics were reported with 95% confidence intervals (CIs). Publication bias was assessed by visual examination of the funnel plots and by using Egger's asymmetry test.

## 3. Results

### 3.1. Included Studies and Patient Characteristics

**Figure 1:**
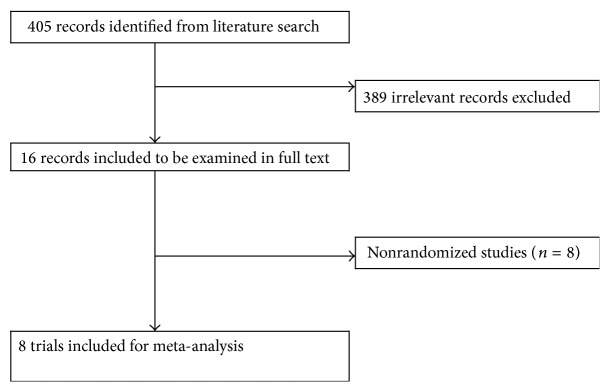
Flow chart of trials selection. Flow chart shows the literature search for RCTs of NDHP versus control group for patients with slow flow or no reflow. RCTs indicate randomized controlled trial.

We initially identified 405 potentially relevant articles and 16 articles were considered to be of interest and were retrieved for full-text review. Eight non-RCTs studies were excluded, and the remaining 8 RCTs studies [16, 19–25] were analyzed.  Figure 1 shows the flow chart of the study selection process. Of the 8 articles, one article [20] reported the results of the RECOVER trial by comparison of three groups (verapamil group, diltiazem group, and control group); we analyzed these data by dividing the data sets into verapamil versus control and diltiazem versus control. The 8 published RCTs included a total of 494 patients (261 allocated to NDHP and 233 to the control group). Study quality was generally low-to-intermediate (three studies with JADAD scale 2, three studies with JADAD scale 3, and only one study with JADAD scale 6).  Table 1 shows the study design and baseline patient characteristics for each of the eligible trials. No significant differences were found in baseline characteristics between the 2 groups. The mean follow-up period ranged from 1 to 6 months.

### 3.2. Outcomes

#### 3.2.1. TIMI Flow

**Figure 2:**
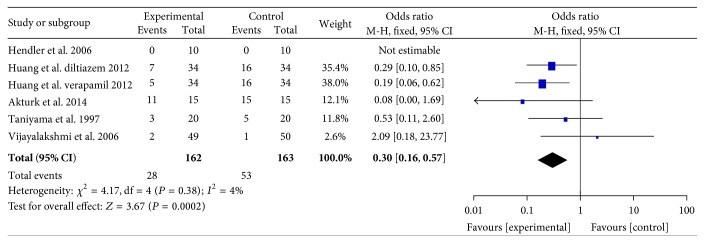
Pooled risk ratio of verapamil/diltiazem versus control for TIMI flow after percutaneous coronary intervention. CI, confidence interval; M-H, Mantel-Haenszel; TIMI, thrombolysis in myocardial infarction.

Data on TIMI flow were available from five studies [16, 19, 20, 22, 25] randomizing 325 participants, of whom 162 received verapamil/diltiazem therapy and 163 received control therapy. The pooling analysis showed that intracoronary verapamil/diltiazem injection significantly decreased the occurrence of the coronary no reflow phenomenon (RR: 0.3, 95% CI: 0.16 to 0.57; *P* = 0.0002, *I* ^2^ = 4%; Figure 2).

#### 3.2.2. CTFC

**Figure 3:**
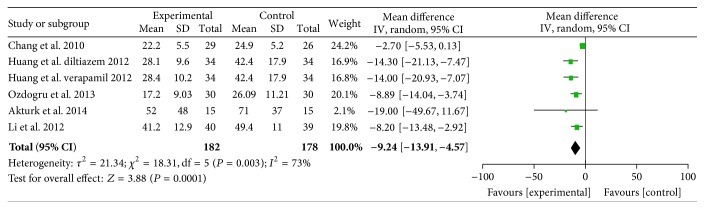
Pooled mean difference of verapamil/diltiazem therapy versus control for correct thrombolysis in myocardial infarction frame count after percutaneous coronary intervention. CI, confidence intervals; IV, inverse variance; SD, standard deviation.

Data on CTFC were available from six data sets derived from five studies [21–25] randomizing 360 participants. Intracoronary verapamil/diltiazem injection or oral diltiazem significantly decreased the CTFC as compared to control (WMD = −9.24, 95% CI −13.91 to 4.57; *P* = 0.0001; *I* ^2^ = 73%, Figure 3).

#### 3.2.3. LVEF

**Figure 4:**
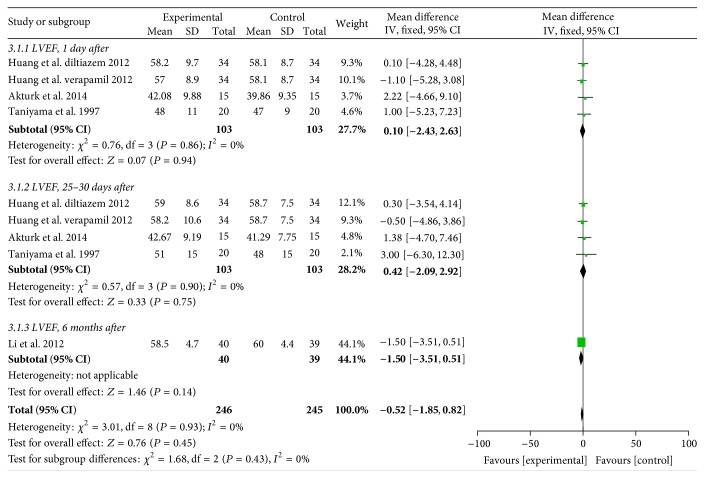
Pooled mean difference of experimental versus control group for left ventricular ejection fraction (LVEF) after percutaneous coronary intervention (PCI). CI, confidence intervals; IV, inverse variance; SD, standard deviation.

Three RCTs [16, 22, 23] reported LVEF values and there was no significant difference on LVEF between NDHP and control groups within one week (WMD, 0.1; 95% CI, −2.43 to 2.63; *P* = 0.94; *I* ^2^ = 0%, Figure 4), and after 30 days (WMD, 0.42; 95% CI, −2.09 to 2.92; *P* = 0.75; *I* ^2^ = 0%, Figure 4). One study [25] observed no difference on LVEF between baseline and 6 months after therapy between oral diltiazem group and control group (*P* > 0.05, Figure 4).

#### 3.2.4. WMI

**Figure 5:**
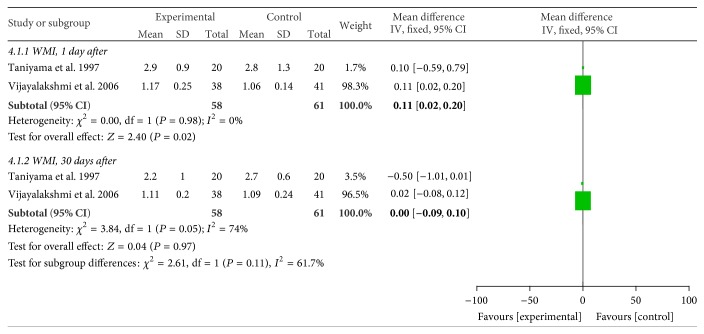
Pooled risk ratio of experimental therapy versus control for WMI after percutaneous coronary intervention. CI, confidence interval; M-H, Mantel-Haenszel.

WMI data were available from two studies [16, 19]; verapamil/diltiazem treatment showed superiority in reducing WMI compared to the control at day 1 (*P* < 0.05, Figure 5). However, there was no significant difference at day 30 after therapy (*P* > 0.05, Figure 5).

#### 3.2.5. MACEs

**Figure 6:**
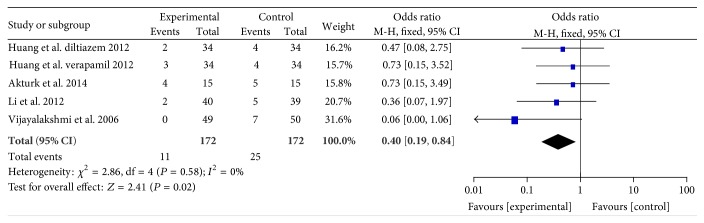
Pooled risk ratio of experimental therapy versus control for major adverse cardiac events after percutaneous coronary intervention. CI, confidence interval; M-H, Mantel-Haenszel.

Five experimental data from four RCTs [19, 22, 23, 25] reported the MACEs. Incidence of MACEs was significantly lower in intracoronary verapamil/diltiazem injection or oral diltiazem groups compared to control group at 6 months after PCI (WMD: 0.4, 95% CI: 0.19 to 0.84; *P* = 0.02; *I* ^2^ = 0%, Figure 6).

### 3.3. Publication Bias

There is no evidence of publication bias for studies included in this meta-analysis based on the Egger's test (*P* = 0.308).

### 3.4. Side Effects

Side effects were not reported in 3 RCTs [20, 21, 25]. No side effects were observed in one study [16]. Vijayalakshmi et al. reported transient blood pressure reduction and heart block up to 3 h after intracoronary verapamil injection in nine patients [19]. Huang and colleagues also observed transient blood pressure reduction (9 mmHg) and bradycardia in patients after intracoronary verapamil injection (4 sinus bradycardia and 5 cases with AVB ≥ 2, 4 out of 5 patients received pacemaker implantation [22]). Li et al. reported reduced heart rate after oral diltiazem treatment [23] and Ozdogru et al. reported heart rate and blood pressure reduction after intracoronary verapamil injection [24]. In general, NDHP use is safe, but heart rate and blood pressure monitoring are essential during NDHP therapy to observe and timely treat the bradycardia and blood pressure reduction when indicated.

## 4. Discussion

Despite intense research in the field of NRP, no single mechanical or pharmacological therapy has demonstrated a clear efficacy against NRP, due to its multifactorial nature [27]. Results from present meta-analysis indicate that intracoronary verapamil/diltiazem injection or oral diltiazem is associated with a significantly improved TIMI flow and CTFC and reduced incidence of short-term MACEs in patients with no/slow flow. In addition, the procedure significantly reduced WMI compared to the control group. However, verapamil/diltiazem treatment did not affect cardiac systolic function as shown by similar LVEF between NDHP and control group.

Verapamil/diltiazem, nondihydropyridine calcium channel blocker, is known to block the L-type channel in the cell membrane and decrease heart rate, the rate of atrioventricular conduction, blood pressure, and myocardial contractility [28, 29]. However, verapamil/diltiazem treatment did not affect cardiac systolic function as shown by similar LVEF between verapamil/diltiazem and control group based on data from this analysis. As a class, verapamil/diltiazem are beneficial in the prevention of the no reflow phenomenon that is thought to be related to their role in coronary vasodilation. Anatomic no reflow, as assessed by thioflavins [30], includes two different forms: structural and functional no reflow. In functional no reflow, patency of microvessels is compromised because of spasm and/or microembolisation instead of structural distintegrigy [31]. Alleviated coronary artery spasm, especially in the distal coronary artery, might be the major potential mechanism of intracoronary verapamil/diltiazem injection on attenuating the NRP [32].

## 5. Clinical Implication

Results from present analysis suggest that NDHP is uniquely effective in attenuating NRP and reducing short-term MACEs in NRP patients and these agents might be safely used in daily clinical work to combat the NRP. It is to note that NDHP use, especially the intracoronary verapamil injection, is sometimes related to blood pressure reduction and bradycardia; thus, blood pressure and heart rate monitoring are needed during the NDHP use. Future studies with large patient cohort are warranted to prove their efficacy and also in comparison with other potential effective medications currently used to manage NRP including antiplatelet therapy, adenosine, nicardipine, nicorandil, and sodium nitroprusside [27].

## 6. Limitations

This meta-analysis has several limitations. First, detailed description on the method of randomization and allocation concealment process of the 8 RCTs trials was not available; thus, we cannot exclude the existence of selection bias which might affect the clinical outcomes between the NDHP and control group. Second, examined parameters such as TIMI flow and CTFC are somehow objective indicators; the measurement bias might thus affect the reported results. Third, the Jadad score for the included 8 RCTs is not high because the treatment assignments are not double-blinded. Therefore, the results of the present analysis need to be interpreted with caution and may warrant further investigation with larger patient cohort and longer observation period.

## 7. Conclusion

NDHP (verapamil/diltiazem) use is safe and beneficial in attenuating NRP and reducing the 6-month MACEs in NRP patients.

## Figures and Tables

**Table 1:** Baseline characteristics of included studies.

Study	Year	*N*	*N*	*N*	Interventions		Outcomes	Jadad score
		(Ver.)	(Dil.)	(Con.)	Expt.	Ctrl.		
Taniyama et al. [16]	1997	20	0	20	0.5 mg verapamil (diluted in 10 mL saline) 120 mg po follow-up study	Blank	TIMI/LVEF/WMI	3
Vijayalakshmi et al. [19]	2006	49	0	50	0.5 mg verapamil	Heparinized	TIMI/MACE/TFC/WMI	3
Hendler et al. [20]	2006	10	0	10	0.5 mg verapamil	Heparinized	TIMI/LVEF	2
Chang et al. [21]	2010	29	0	35	100–400 *μ*g verapamil	Nitroglycerin	CTFC	2
Huang et al. [22]	2012	34	34	34	200 *μ*g verapamil (max 1 mg) 400 *μ*g diltiazem (max 1 mg)	Nitroglycerin (max 1 mg)	TIMI/CTFC/LVEF/MACE	3
Li et al. [23]	2012	0	40	39	Diltiazem 90 mg bid/6 months	Placebo	TIMI/CTFC/LVEF/MACE	6
Ozdogru et al. [24]	2013	0	30	30	5 mg diltiazem	Nitroglycerin	CTFC/HR/BP	2
Akturk et al. [25]	2014	15	0	15	Verapamil	Placebo	CTFC/LEVF	2
Overall		157	104	233				
